# Molecular Genetics and Reproductive Genomic Medicine in Cleft Lip and Palate: Current Evidence, Preimplantation Testing, and Clinical Translation—A Narrative Review

**DOI:** 10.3390/ijms27208980

**Published:** 2026-10-10

**Authors:** Rui Fan, Haofei Shen, Yaru Liu, Tingting Ji, Lili Zhang, Xiaoling Ma

**Affiliations:** 1 The First Clinical Medical College, Lanzhou University, Lanzhou 730000, China; fanr2025@lzu.edu.cn (R.F.); 715106922@163.com (H.S.);; 2 Department of Reproductive, First Hospital of Lanzhou University, Lanzhou 730000, China; 3 Clinical Research Center for Reproductive Diseases of Gansu Province, Lanzhou 730000, China

**Keywords:** cleft lip and palate, orofacial clefts, preimplantation genetic testing, polygenic risk scores, reproductive ethics

## Abstract

Cleft lip and palate (CLP) is a common congenital craniofacial anomaly with heterogeneous clinical manifestations and complex etiologies. Current evidence indicates that CLP arises from the combined effects of Mendelian syndromic disorders, rare pathogenic variants, common susceptibility loci, non-coding regulatory variants, epigenetic regulation, mitochondrial dysfunction, and maternal environmental exposures. Although reconstructive surgery and multidisciplinary care remain central to treatment, families with recurrent CLP or a family history of orofacial clefts increasingly seek reproductive counseling and genetic risk assessment before pregnancy. Preimplantation genetic testing (PGT) may provide a clinically meaningful option when a pathogenic variant, chromosomal abnormality, or clearly defined familial haplotype is identified. However, most non-syndromic CLP cases are polygenic or multifactorial, and the application of preimplantation genetic testing for polygenic disorders (PGT-P) remains limited by modest predictive accuracy, population-specific transferability, embryo-number constraints, incomplete penetrance, variants of uncertain significance, and uncertain long-term clinical utility. This narrative review summarizes the clinical phenotypes and genomic architecture of CLP, evaluates the principles and current limitations of preimplantation genetic testing for monogenic disorders (PGT-M) and PGT-P, and discusses psychosocial and ethical issues in reproductive decision-making. We propose that PGT for CLP should be positioned as part of a stepwise counseling framework rather than as a universal preventive strategy. Future progress will depend on standardized phenotyping, multi-ancestry genomic databases, functional validation of candidate variants, transparent risk communication, and shared decision-making that respects both reproductive autonomy and the uncertainty of genomic prediction.

## 1. Introduction

Cleft lip and palate (CLP) is one of the most common congenital malformations [1], affecting approximately one in 700 live births globally, with substantial variability across populations and geographic regions [2,3]. As a structural anomaly of the lip and/or palate [1], CLP arises from the disruption of normal facial development during early embryogenesis, typically within the first nine weeks of gestation. Its phenotypic spectrum extends beyond anatomic clefts to encompass a range of secondary complications, including dental malformations, maxillofacial growth deficiencies, speech impairment, hearing loss, and psychosocial challenges [4,5,6,7]. These multifactorial clinical consequences highlight CLP as not merely an isolated craniofacial defect but a complex developmental disorder that affects overall health and quality of life. From a genetic perspective, CLP represents a prototypical example of a multifactorial birth defect influenced by both genetic predisposition and environmental exposure. Whole-exome sequencing (WES) and integrative multi-omics analyses have revealed that both common and rare variants contribute to risk, yet no single mutation explains most cases, particularly in non-syndromic forms. Furthermore, maternal health conditions such as obesity, diabetes, hypertension, and tobacco exposure interact with genetic susceptibility to modulate disease risk [8]. These findings reinforce the necessity of understanding CLP as a genetically heterogeneous disorder with incomplete penetrance and variable expressivity. Clinically, individuals with CLP experience considerable functional and psychosocial burdens. Infants with CLP often require early feeding interventions and multiple reconstructive surgeries throughout childhood and adolescence. Beyond medical care, the stigma associated with orofacial cleft may lead to social isolation, depression, and decreased life satisfaction, particularly in resource-limited settings. As a result, preventing recurrence through precise genetic diagnosis and reproductive counseling has become a critical focus in both clinical genetics and public health.

Reproductive medicine offers new opportunities for families affected by hereditary CLP [9]. Preimplantation genetic testing (PGT), integrated with In Vitro Fertilization (IVF), enables the identification of embryos carrying pathogenic variants before implantation, thus potentially reducing the likelihood of recurrence. Studies using WES and whole-genome sequencing (WGS) have identified rare pathogenic variants associated with CLP, suggesting that PGT could theoretically prevent the transmission of known mutations [10]. However, this approach faces major limitations: most CLP cases are polygenic or multifactorial rather than monogenic, variant interpretation remains uncertain, and environmental modifiers cannot be fully controlled. Not all genetic abnormalities in patients with CLP can be accurately detected by current technologies. For the more common non-syndromic cleft lip and palate, even with the most advanced WGS technology, genetic abnormalities are still indeterminate, probably because the genetic contributions are still not explained completely. These factors also pose challenges for the screening and diagnosis of CLP. Consequently, PGT for CLP remains largely experimental and ethically debated. Beyond the laboratory, reproductive decision-making for CLP-affected families involves complex psychological and ethical dimensions. Couples must navigate uncertainty regarding genetic results, weigh medical recommendations against personal or cultural values, and cope with potential emotional distress. Shared decision-making frameworks, emphasizing transparent communication between clinicians and patients, are increasingly recognized as essential in guiding reproductive choices [11]. Such approaches promote autonomy and informed consent while addressing the emotional impact of genetic uncertainty. Shared decision-making can reduce decision conflicts and improve risk perception. Healthcare providers should discuss reproductive options with couples who have a history of CLP but desire fertility. This should include clearly explaining the genetic probabilities, identifying the types, and estimating the recurrence risks to fully respect the patient’s decision autonomy. In principle, genetic testing can be conducted for all related patients or individuals who have a family history of the condition but normal phenotypes. However, considering that the genetic background is not yet fully understood and the causes are complex, a negative result from genetic testing does not rule out the risk of offspring developing CLP. Therefore, when making clinical decisions, it is important to comprehensively collect the patient’s medical history to make a comprehensive risk assessment [11].

In this narrative review, we summarize the clinical phenotypes and genomic architecture of CLP, with particular emphasis on the genetic heterogeneity, incomplete penetrance, and gene–environment interactions that limit risk prediction. In addition to the commonly used preimplantation genetic testing for aneuploidy (PGT-A), preimplantation genetic testing for monogenic disorders (PGT-M), and preimplantation genetic testing for structural rearrangement (PGT-SR), preimplantation genetic testing for polygenic risk (PGT-P) is an emerging and promising tool for polygenic risk estimation. It is important to distinguish these modalities: PGT-M detects a defined familial pathogenic variant and yields a deterministic diagnosis, whereas PGT-P provides only a probabilistic ranking of embryos based on polygenic risk scores and does not constitute a diagnostic result. Finally, we discuss the psychosocial and ethical challenges involved in reproductive decision-making. By integrating evidence from clinical genetics, reproductive medicine, and bioethics, this review aims to provide a practical framework for genetic counseling and clarify the future directions required for the responsible clinical translation of reproductive genomic technologies in CLP.

## 2. Clinical Phenotypes and Genomic Architecture of CLP

### 2.1. Epidemiology and Phenotypic Classification

Orofacial clefts (OFCs), encompassing cleft lip only (CLO), cleft palate only (CPO), and CLP, represent the most prevalent congenital anomalies of the craniofacial region and the second most common birth defect worldwide [1,2]. Their global birth prevalence is estimated at 1 in 700 live births, though incidence varies markedly by geography, ethnicity, and socioeconomic status [1,3,12]. Studies have consistently reported higher rates in Asian and Latin American populations, intermediate rates in Europeans, and lower frequencies in individuals of African ancestry [13]. Within these subtypes, CPO typically occurs less frequently than CLP, and sex distribution differs between forms—males are more likely to have CLP, whereas females are more prone to isolated CPO [13]. The classification of CLP is essential for both clinical management and genetic analysis. From an anatomic standpoint, clefts may involve CLO, CLP, or CPO. Rare craniofacial clefts that extend beyond the typical orofacial regions form an additional category [5]. CLO and CLP are often grouped together as “CLP” due to shared developmental pathways. From a genetic and clinical perspective, OFCs are broadly categorized as syndromic orofacial cleft (SOFC) or non-syndromic orofacial cleft (NSOFC) [1,14]. Syndromic cases, accounting for roughly 30% of CLP and 50% of CPO cases, are associated with additional systemic anomalies or recognizable genetic syndromes such as Van der Woude syndrome or Pierre Robin sequence [14,15]. In contrast, NSOFCs are typically isolated, sporadic, and result from complex gene–environment interactions rather than single-gene defects [1]. Precise classification is fundamental not only to predict recurrence risk but also to facilitate international collaboration and standardization in research. Phenotypic subtyping, such as laterality (unilateral/bilateral) and completeness (complete/incomplete), further refines diagnosis and helps correlate clinical presentation with genetic background [14,16]. These detailed taxonomies also assist in surgical planning and prognostic counseling.

### 2.2. Clinical Manifestations and Multidisciplinary Comorbidities

Although CLP is rarely life-threatening, its functional and aesthetic consequences are profound. The malformation affects feeding, speech, hearing, dentition, and psychological development, often requiring a multidisciplinary care approach. Craniofacial surgeons, orthodontists, speech pathologists, and psychologists typically coordinate lifelong management [17]. Dental abnormalities are among the most common secondary consequences of CLP [18]. These include malocclusion, tooth agenesis, microdontia, macrodontia, supernumerary teeth, ectopic canines, and enamel hypoplasia. A meta-analysis involving 27,703 patients across 34 countries found that individuals with non-syndromic cleft lip and/or palate (NSCL/P) exhibit a significantly higher risk of tooth anomalies in both deciduous and permanent dentition compared with healthy controls [18]. Hypodontia and tooth malposition are particularly frequent, often localized near the cleft margins. One recent cross-sectional study elucidated that children with CLP have delayed dental development and eruption compared with healthy children [19]. Furthermore, CLP alters oral ecology and increases susceptibility to dental caries and periodontal disease, likely due to structural irregularities and difficulties in maintaining oral hygiene [20]. Patients commonly display maxillary growth deficiencies, leading to malocclusion and midface retrusion. TP63-related syndromes can present with a high incidence of hypoplasia and missing permanent maxillary incisors [21]. Muscular dysfunction of the orbicularis oris has been linked to cytochrome coxidase deficiency, a mitochondrial defect that impairs muscle energy metabolism and contributes to abnormal lip development [22]. Such findings suggest mitochondrial involvement in certain CLP phenotypes, expanding the classical nuclear-gene-centric understanding of the disorder. Beyond the craniofacial phenotype, CLP patients may present with a broad spectrum of extracranial manifestations. Otitis media with conductive hearing loss frequently occurs secondary to palatal muscle insufficiency [23]. Syndromic presentations encompass multisystem involvement, including cutaneous abnormalities (such as skin fragility, sparse or wiry hair), ocular anomalies (e.g., ankyloblepharon, lacrimal duct obstruction, and corneal erosions), and limb defects such as syndactyly [24,25]. Cardiovascular septal defects are among the most frequently co-occurring systemic anomalies [26,27], and respiratory compromise with feeding difficulties may require gastrostomy placement in infancy. Notably, certain OFC are associated with immune dysregulation (such as autoimmune lymphoproliferative syndrome and T-cell lymphopenia), and experimental evidence has linked TP63 deficiency to immune system disorders [25,28,29,30]. These observations suggest that the immunological pathway may represent a promising avenue for further exploration of genotype–phenotype correlations. Collectively, these syndromic features underscore the importance of thorough multisystem evaluation and highlight the need for precise phenotypic delineation to guide genetic testing strategies and inform reproductive risk counseling.

Beyond physical health, the psychosocial burden of CLP is substantial. Children often experience social stigma and bullying, leading to reduced self-esteem, anxiety, and depression [31]. These issues are particularly acute in low- and middle-income countries (LMICs), where access to reconstructive surgery [32] and psychosocial support [33] remains limited. Consequently, addressing both functional and emotional well-being is essential in holistic CLP care.

### 2.3. Molecular Pathogenesis and Etiological Complexity

**Figure 1:**
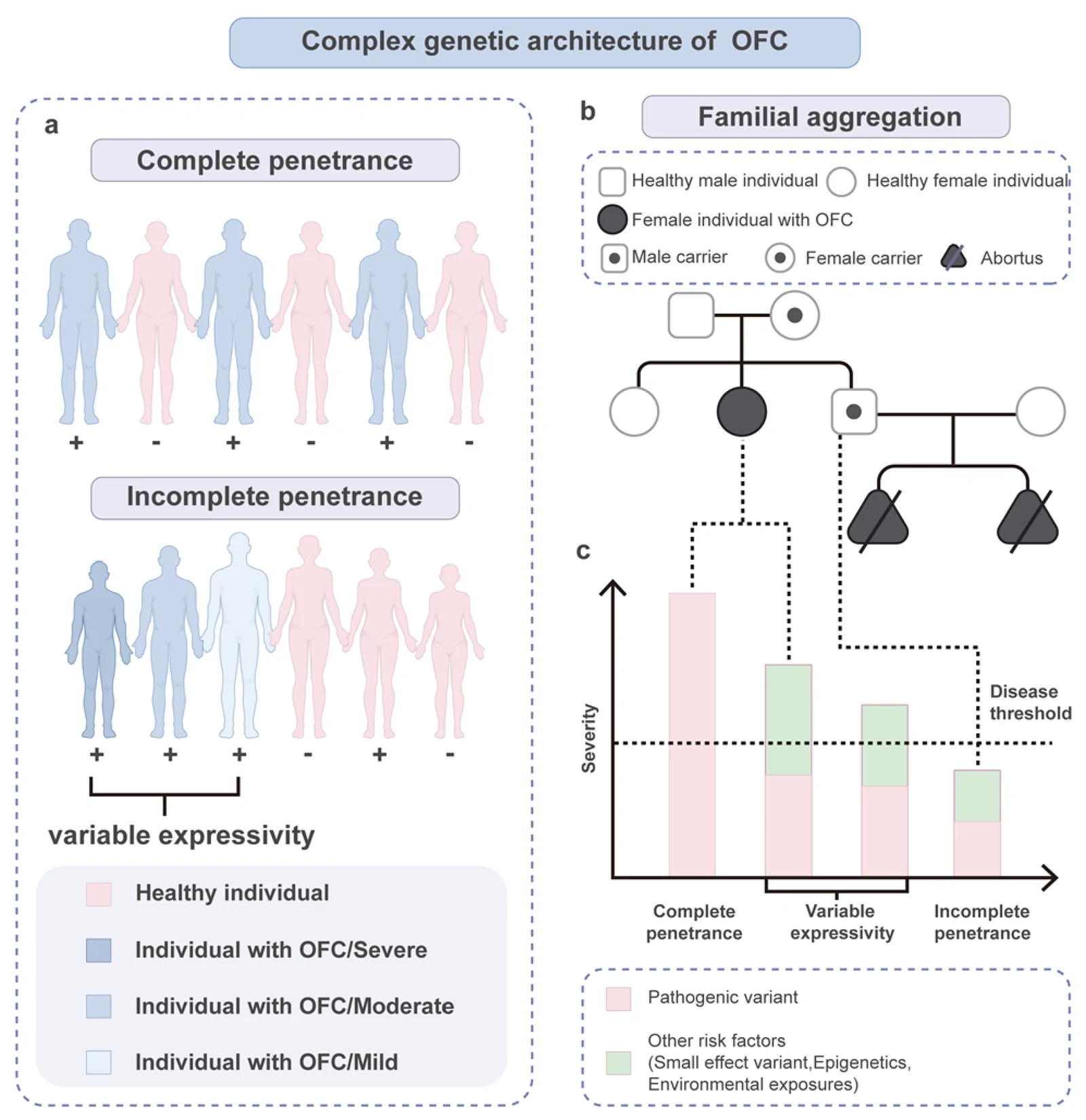
Complex genetic architecture of orofacial cleft (OFC). (**a**) In the condition of complete penetrance, every individual with a pathogenic variant (marked as +) shows this disease. Individuals without carrying any pathogenic variants are all marked as −. In the condition of incomplete penetrance, some individuals with pathogenic variants cannot develop this disease. Because of variable expressivity, even the same pathogenic variant can lead to varying degrees of disease. (**b**) In some family cases with a family aggregation of OFC, some individuals with the same inherited pathogenic variant show normal manifestation. (**c**) Besides pathogenic variant, there are other risk factors, including small-effect variants, epigenetics, and environmental exposures. These cumulative effects lead to incomplete penetrance and variable expressivity.

The pathogenesis of CLP reflects the disruption of precisely coordinated molecular and cellular processes during embryonic facial morphogenesis. Palatogenesis involves temporospatially regulated cellular migration, proliferation, and fusion of the facial prominences [4,34]. Aberrations in any of these processes, triggered by genetic mutations or environmental insults, can lead to orofacial clefts. In other words, based on this complex context, we can roughly divide CLP into developmental and genetic origins (Figure 1).

Genetic studies have identified numerous loci associated with CLP risk. The *IRF6* gene, which encodes interferon regulatory factor 6, plays a central role in epithelial differentiation and is implicated in both syndromic and non-syndromic forms [35,36]. Mutations in *MSX1*, *HIF3* and *FGFR1* have also been linked to defective craniofacial patterning [37,38,39,40,41,42,43,44,45]. Besides syndromic disorders, certain TP63-related OFC follow an autosomal dominant pattern. More recent research highlights regulatory networks involving FGF, WNT, and TGF-β signaling pathways as crucial to palatal fusion [46]. A new report revealed that antagonism of the signal transducer Smoothened would cause OFCs by disrupting the sonic hedgehog (SHH) pathway, and this mechanism has a dose–response relationship with the incidence of OFC [47]. The exposure windows of signaling pathway disruption also influence adverse outcomes. The disruptions lead to abortion early in gestation and facial prominences or OFC later in development [47]. The integration of exome and genome sequencing data continues to expand the catalog of candidate genes, suggesting that rare variants collectively contribute to disease risk rather than a single causative mutation.

Beyond coding mutations, epigenetic modifications and non-coding RNAs significantly influence craniofacial development. DNA methylation, histone modification, and the activity of long non-coding RNAs (lncRNAs) and microRNAs (miRNAs) regulate the expression of cleft-related genes [48,49,50]. For example, CpG-SNPs have been associated with non-syndromic CLP in certain populations [49], while studies of monozygotic twins discordant for Van der Woude syndrome reveal methylation differences that modulate phenotypic variability [50]. Such findings highlight the intricate interplay between genome and epigenome in shaping developmental outcomes. Previous evidence implicates mitochondrial DNA (mtDNA) abnormalities being related to CLP pathogenesis [51]. Emerging evidence has verified this point and proposed a more detailed mechanism. Defects in oxidative phosphorylation, such as cytochrome c oxidase deficiency, disrupt muscle energy metabolism and contribute to lip and palate malformation [22]. Additionally, large-scale trio sequencing studies have identified de novo protein-altering variants that may distinguish syndromic from non-syndromic CLP [52,53]. Because de novo variants are not subject to traditional selective pressures, they represent an important reservoir for pathogenic alleles in sporadic cases.

Maternal health and environmental exposures critically modulate genetic susceptibility. Factors such as smoking, obesity, hypertension, diabetes, maternal diet (such as the Western diet), and nutritional deficiencies increase CLP risk, potentially through oxidative stress and inflammatory mechanisms [8].

Maternal metabolic conditions can alter the fetal epigenome through aberrant DNA methylation, histone modifications, and dysregulated microRNA expression, thereby affecting the activity of morphogenic signaling pathways such as WNT that are essential for craniofacial development, ultimately contributing to the pathogenesis of CLP [54]. In a systematic review, Sun et al. synthesized the evidence in this field and suggested this point [54]. A recent narrative review published in *Frontiers in Public Health* in 2025 further corroborated these findings, demonstrating that gestational diabetes mellitus and maternal obesity may elevate the risk of CLP through inflammatory responses, oxidative stress, and impaired craniofacial development in the fetus [55]. Unequal placental sharing (as in monozygotic twins) [56], disparities in vascular perfusion, and oxygen delivery could also lead to OFC. Moreover, Blanco et al. demonstrated that maternal hyperhomocysteinemia is significantly associated with an increased risk of NSOFC, highlighting the role of aberrant methyl donor intake or metabolism in the pathogenesis of cleft lip and palate [57]. Albu et al. systematically reviewed the role of folic acid in oral health and concluded that folate deficiency contributes to the development of OFC through mechanisms involving genomic instability, aberrant DNA methylation, and oxidative stress [58].

Environmental teratogens like retinoic acid (RA) and dioxins (TCDD) have also been shown to disrupt neural crest cell migration and palatal fusion [59]. The effects of medications, including nonprescription ones, need to be monitored [60]. Cigarette smoke (such as active or passive smoking) has been shown to affect palatal development through multiple pathways: (1) carbon monoxide (CO) inducing placental hypoxia; (2) nicotine-mediated vasoconstriction of uterine arteries leading to embryonic tissue hypoxia; (3) polycyclic aromatic hydrocarbons (PAHs) combining with the aryl hydrocarbon receptor (AHR); and (4) disruption of DNA methylation patterns [61]. Ács et al. reported that maternal smoking increases the risk of CL/P by approximately 1.58-fold, with passive smoke exposure conferring an even higher risk than active smoking [8]. With respect to medications and teratogens, antiseizure medications such as valproic acid are known to influence gene expression through inhibition of histone deacetylases (HDACs), thereby contributing to teratogenic effects [62]. Finally, concerning environmental and occupational pollutants, heavy metals including arsenic and lead may affect DNA methylation status via inhibition of DNA methyltransferases (DNMTs) and induction of oxidative damage. Exposure to gaseous pollutants such as CO, nitrogen dioxide (NO_2_), and sulfur dioxide (SO_2_), as well as particulate matter (PM_2.5_), has been associated with an elevated risk of OFC [61]. Specifically, a 10 μg/m^3^ increase in PM_2.5_ concentration corresponds to a 43% increase in the risk of CPO [63].

Such evidence underscores that CLP cannot be fully explained by genetics alone but arises from multifactorial etiologic interactions.

### 2.4. Limitations in Risk Stratification and Clinical Translation

The genetic landscape of CLP is characterized by heterogeneity, polygenic inheritance, and variable expressivity. While syndromic forms follow recognizable Mendelian patterns, non-syndromic CLP involves multiple loci [64]. This complexity complicates both gene discovery and clinical translation. Many individuals carrying risk alleles do not manifest clefts, reflecting incomplete penetrance and the influence of modifier genes. Epigenetic factors, such as differential DNA methylation and chromatin remodeling, further modulate phenotypic outcomes [48,49]. Multi-omics integration, combining genomic, transcriptomic, and epigenomic data, is thus essential to unravel these interactions [43,65]. Despite progress, current predictive models remain limited. Polygenic risk scores (PRSs) have been applied to estimate CLP susceptibility, but their predictive accuracy is modest due to the dispersed and context-dependent nature of risk alleles [66]. Unlike monogenic disorders, CLP’s polygenic structure defies simple diagnostic categorization, making personalized reproductive prediction challenging. Bridging the gap between gene discovery and clinical intervention remains a central challenge. While sequencing studies have identified numerous loci, few have yielded actionable targets for prevention or prenatal diagnosis. The complexity of gene–environment interactions and population-specific variation further limits generalizability [67]. To improve clinical relevance, future research must focus on larger, multi-ethnic cohorts and standardized phenotyping to enhance statistical power and cross-population applicability.

In summary, CLP represents a paradigm of multifactorial inheritance involving both nuclear and mitochondrial genomes, epigenetic modulation, and environmental interaction. Although remarkable progress has been made in identifying candidate genes and mechanisms, translating this knowledge into effective risk prediction and prevention strategies remains elusive. Understanding these molecular underpinnings lays the groundwork for advanced reproductive technologies and personalized genetic counseling.

## 3. Advances in Reproductive Genomic Technologies

### 3.1. Advances in Molecular Mechanisms and Multi-Omics Research

The evolutionary trajectory from Sanger sequencing to next-generation sequencing (NGS) and third-generation sequencing (TGS), and then to epigenomics, single-cell sequencing (SCS), spatial omics, and multi-omics integration, reveals that research on OFC has shifted from susceptibility mapping to mechanistic elucidation [68].

To date, GWASs have identified over 40 susceptibility loci for NSCL/P. However, the susceptibility variants within these loci and their functional roles remain largely undefined [69]. Therefore, precise fine-mapping of these susceptibility variants and in-depth investigation into the underlying biological mechanisms are essential for advancing our understanding of the genetic etiology of NSCL/P. Notably, a subset of these variants are enriched in or near regulatory elements, such as enhancers and promoters, and may contribute to disease risk by modulating the functional activity of these elements, thereby altering gene expression patterns. New research has discovered that among 66 de novo variants (DNVs), 17 (25.7%) were within a predicted promoter region. Two DNVs located within the same enhancer region (hs1617) showed significant enrichment above expectation by chance (*p* = 0.0017) and were predicted to affect the binding of transcription factors such as PAX6 and ZBTB7A. Notably, this enhancer region resides within the same topologically associating domain (TAD) as genes involved in craniofacial development, including *HHAT* [70]. In another study, conditional analysis of GWASs suggested that two functional single nucleotide polymorphisms (SNPs) near *IRF6* account for the majority of risk for CL/P associated with variation at this locus [37].

The advancement of SCS technologies has enabled the dissection of cellular heterogeneity in craniofacial tissues during early embryonic development. A single-nucleus RNA sequencing (snRNA-seq) study of craniofacial tissues from 28 human embryos (4–8 weeks of gestation) systematically identified eight major cell types and reconstructed cellular differentiation trajectories. We have learned that de novo protein-truncating variants in patients with orofacial clefts are significantly enriched in the gene expression networks of specific ectodermal and epithelial subtypes, suggesting differential contributions of distinct cell lineages to the pathogenesis of CLP [71]. At the level of spatial transcriptomics, researchers performed the first single-cell spatial RNA sequencing study in a *Pax9* knockout mouse model of CLP, which revealed a synergistic interaction between dysregulated Wnt signaling effectors and disruption of extracellular matrix gene expression [72].

### 3.2. Principles and Clinical Utility of PGT

These disorders (such as Fraser Syndrome [73], Meckel–Gruber Syndrome [74], Gorlin-Goltz Syndrome [75], Hartsfield Syndrome [44], Stickler Syndrome [76], Hay–Wells Syndrome [21], and Fryns Syndrome [77]) are caused by mutations in single genes. For such monogenic disorders with a known causative variant, PGT is an established and effective approach, but inefficient in non-Mendelian disorders or polygenic disorders [78], including epigenetic modifications, post-translational modifications, and even environmental contributions. Early studies have suggested that polygenic risk scores derived from genome-wide genotyping may enable the estimation of disease risk in embryos, with some authors proposing that this approach could achieve risk prediction performance approaching that of monogenic testing in certain contexts [79]. However, it is important to emphasize that PGT-P provides probabilistic risk estimates rather than deterministic diagnostic results, and its clinical utility remains unproven, particularly for complex traits such as CLP. For clarity, detection of a defined familial pathogenic variant is the remit of PGT-M and yields a deterministic result, whereas PGT-P generates probabilistic risk rankings based on polygenic risk scores and should not be interpreted as diagnostic, given the limitations of PRS transferability across ancestral populations, the small number of embryos available for selection, and the confounding influence of environmental and lifestyle factors. PGT has also been explored for maternally inherited mtDNA mutations, although it can only reduce the risk of transmitting a high mutation load rather than eliminate it [80]. Moreover, although we can decrease the influence of gender-related mechanisms through gender selection, there is still a need for further discussion of ethical rationality and clinical practicability.

**Figure 2:**
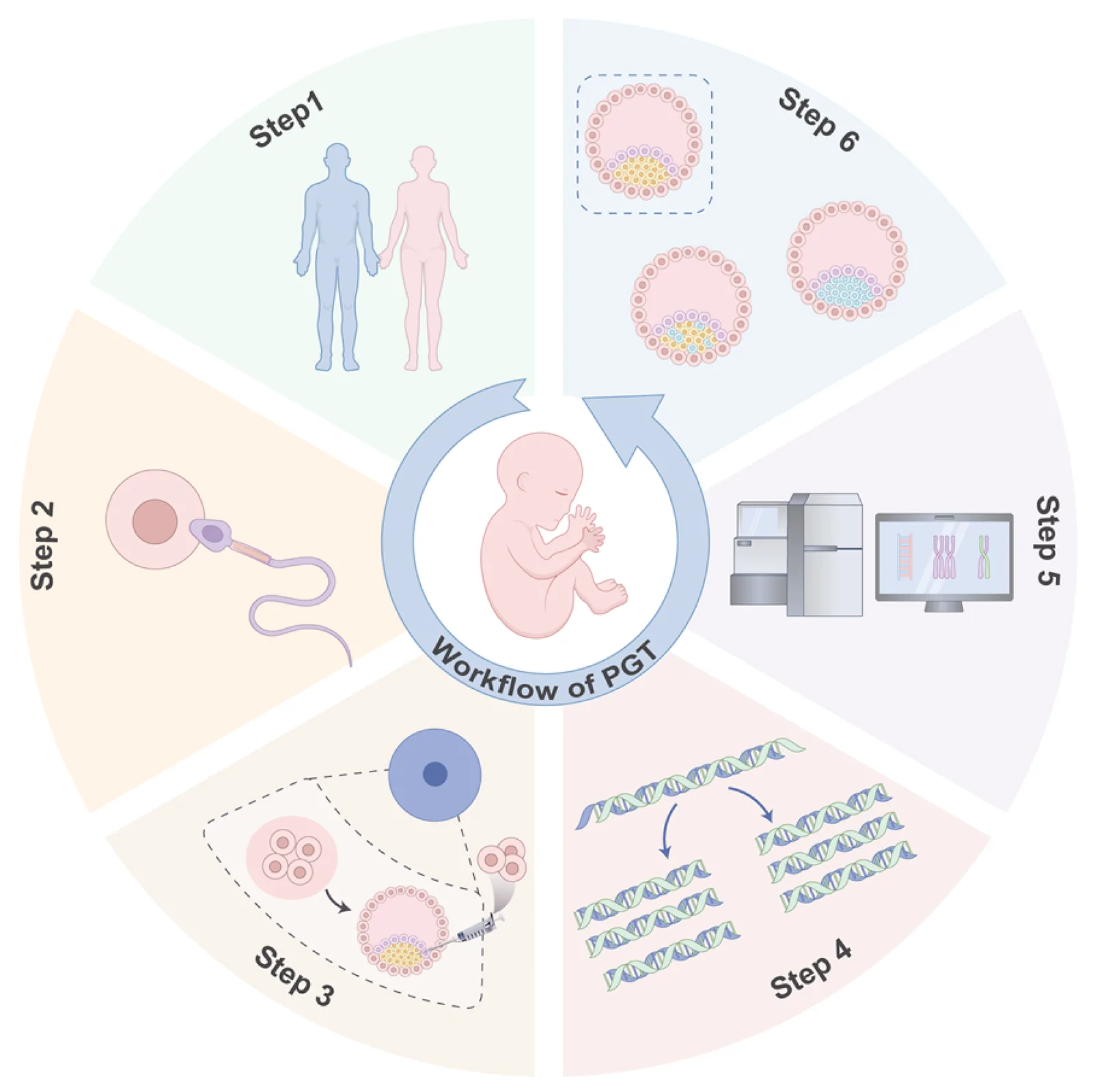
Conventional preimplantation genetic testing (PGT-A, PGT-M, and PGT-SR) workflow. (Step 1) The couple receives genetic counseling and a clear detection target (aneuploidy for PGT-A, a monogenic variant for PGT-M, or a structural rearrangement for PGT-SR) and formulates a personalized plan. (Step 2) IVF and embryo culture using Intracytoplasmic Sperm Injection (ICSI) are carried out, obtaining multiple oocytes through medicine-related ovulation induction and oocyte retrieval, and selected spermatozoa are injected into one egg. (Step 3) Until the blastocyst stage, from day 5 or day 6, 5–10 trophectoderm cells are extracted and a trophectoderm biopsy is performed. (Step 4) DNA amplification, usually using whole-genome amplification (WGA), is conducted. For PGT-M, targeted Polymerase Chain Reaction (PCR) of the causative locus is additionally carried out. (Step 5) Genetic analysis, the analytic platform is selected according to the indication. (Step 6) The results are interpreted; embryo selection and cryo-embryo transfer (CET) are conducted. This workflow applies to PGT-A, PGT-M, and PGT-SR, which yield deterministic results. PGT-P, which provides probabilistic polygenic risk ranking rather than a diagnostic result, is not depicted in this figure.

Conventional PGT workflow operates within a highly coordinated IVF framework (Figure 2). Following genetic counseling to define targets, the process involves controlled ovarian hyperstimulation and oocyte retrieval. Fertilization is typically achieved via Intracytoplasmic Sperm Injection (ICSI), followed by embryo culture to the blastocyst stage (Day 5 or 6). A biopsy of 5–10 trophectoderm cells is then subjected to whole-genome amplification and genetic analysis. The embryos are cryopreserved (vitrification) pending result and are selected thereby reducing the likelihood of inheritance of pathogenic variants.

### 3.3. PRS: Methodological Shifts and Clinical Feasibility

While PGT-M addresses Mendelian disorders, the majority of cleft cases are NSCL/P and polygenic. This has spurred the development of PGT-P. With the recent introduction of PGT-P, a kind of method of risk assessment rather than diagnosis, based on millions of gene loci from whole-genome sequencing data extracted from embryo biopsy cells through large-scale GWAS, the relative risk of embryos developing common complex diseases like NSCL/P can be evaluated in detail by calculating the PRS [79,81]. Thus, we can prioritize embryos with a lower estimated polygenic risk within the available embryo cohort. Preliminary research indicates that this technique is feasible [79], but its clinical effectiveness and long-term outcome still need to be further validated. PGT-P is primarily aimed at screening for serious polygenic diseases. However, this technology is still in the research and exploratory stages, with only preliminary clinical practice and no large-scale adoption to date. Recently, a proof-of-concept study demonstrated the feasibility of improving polygenic risk prediction by incorporating family pedigree information, and used this approach to select the lowest-risk embryo for type 2 diabetes in a single case [82]. Although some limitations exist, such as a small sample size, this research has laid the foundation for future larger-scale and multi-center PGT-P studies. Differences in global application reflect differing regulatory environments and the ongoing debate regarding the clinical utility of PRSs across diverse populations [83]. Overall, the development of PGT-P remains an investigational approach with uncertain clinical utility.

### 3.4. Technological Constraints and Diagnostic Uncertainties

High genetic heterogeneity, complex genotype–phenotype correlations, and high rates of VUS make the interpretation of CLP difficult, emphasizing the need for enhanced international data sharing, more comprehensive functional research and literature support, and better understanding of genotype–phenotype correlations.

In reality, patients with OFC are usually informed about the possibility of false-positive or false-negative results from PGT. A recent review concluded that sound evidence on the obstetric, neonatal, and long-term health outcomes following embryo biopsy for PGT remains limited and inconclusive [84]. Thus, we should emphasize the importance of improved patient counseling, particularly regarding the limitations of PGT, and standard prenatal care regardless of whether PGT was performed.

In terms of ethics, there are challenges in the application and popularization of new technology that is suitable for NSCL/P. The development of PGT-P is accompanied by significant ethical controversy on whether it could be applied to screen for non-disease characteristics like height and intelligence quotient [85]. There is no global consensus on scientific ethics or a supervisory framework for PGT-P. So far, many countries and academic institutions strictly restrict its application, and some countries, like the UK, even stipulate that PGT-P is illegal [83].

Furthermore, models should be improved to address the challenges of PGT because the results, such as risk scores, are greatly influenced by environment, lifestyle, and race [81], which is something that has been overlooked in PGT-P [79]. In addition, its utility relies heavily on the availability of sufficient embryos and clear family histories as evaluation metrics, which may result in some rare monogenic variations being missed. Complex risk scores also pose challenges for genetic counseling and decision-making.

Taken together, although PGT is a novel development in reproductive medicine, further advances in predictive model optimization, the establishment of strict ethical norms, and the development of clear clinical guidelines are needed before wider acceptance can be achieved.

## 4. Psychosocial Impacts and Ethical Frameworks in Reproductive Decision-Making

The integration of advanced reproductive technologies into the management of hereditary CLP has shifted the clinical landscape from purely anatomical repair to preemptive genetic selection. While technologies such as PGT offer the promise of reducing disease recurrence, they simultaneously introduce profound psychosocial burdens and ethical complexities. The shift from testing for high-penetrance monogenic variants to screening for the PRS in embryos—often termed PGT-P—has intensified these debates, raising questions about equity, informed consent, and the definition of a “healthy” life.

### 4.1. Psychosocial Burden and Socio-Cultural Disparities

The diagnosis of a congenital craniofacial anomaly does not merely represent a medical event; it constitutes a profound psychological and socioeconomic crisis for the family unit. The burden of CLP is multidimensional, encompassing the immediate shock of diagnosis, the chronic stress of longitudinal surgical management, and the pervasive anxiety regarding social integration.

In the context of “quality of life” (QoL), families face the dual challenge of managing the stigma associated with orofacial cleft and the rigorous demands of treatment protocols. Restorative management is not a singular event but a trajectory spanning roughly two decades, involving primary lip/palate repair, alveolar bone grafting, orthodontic realignment, and often, complex orthognathic surgery or dental implant placement in adulthood. For parents, this “therapeutic odyssey” imposes severe psychological distress, manifesting as chronic anxiety, guilt, and depression. In LMICs, where public health coverage for secondary revisions and speech therapy is often nonexistent [86], this burden is inextricably linked to economic catastrophe. The financial toxicity of treatment can drive families into poverty, further exacerbating the psychosocial isolation of the affected child.

For couples with a family history of CLP, reproductive decision-making is fraught with emotional turbulence, representing a profound psychological shift from considering pregnancy termination to embracing embryo selection as a preventive strategy. Historically, one important prevention option following prenatal diagnosis via ultrasound was the termination of pregnancy (TOP) [87]. This option carries a heavy psychological toll, often resulting in long-term grief and trauma, particularly in cultures where abortion is stigmatized or legally restricted. In this context, PGT offers an alternative that allows for risk reduction prior to implantation, which some prospective parents may perceive as psychologically different from prenatal diagnosis and termination. Studies suggest that some public view PGT not as “playing God,” but as a responsible parenting strategy [88].

**Figure 3:**
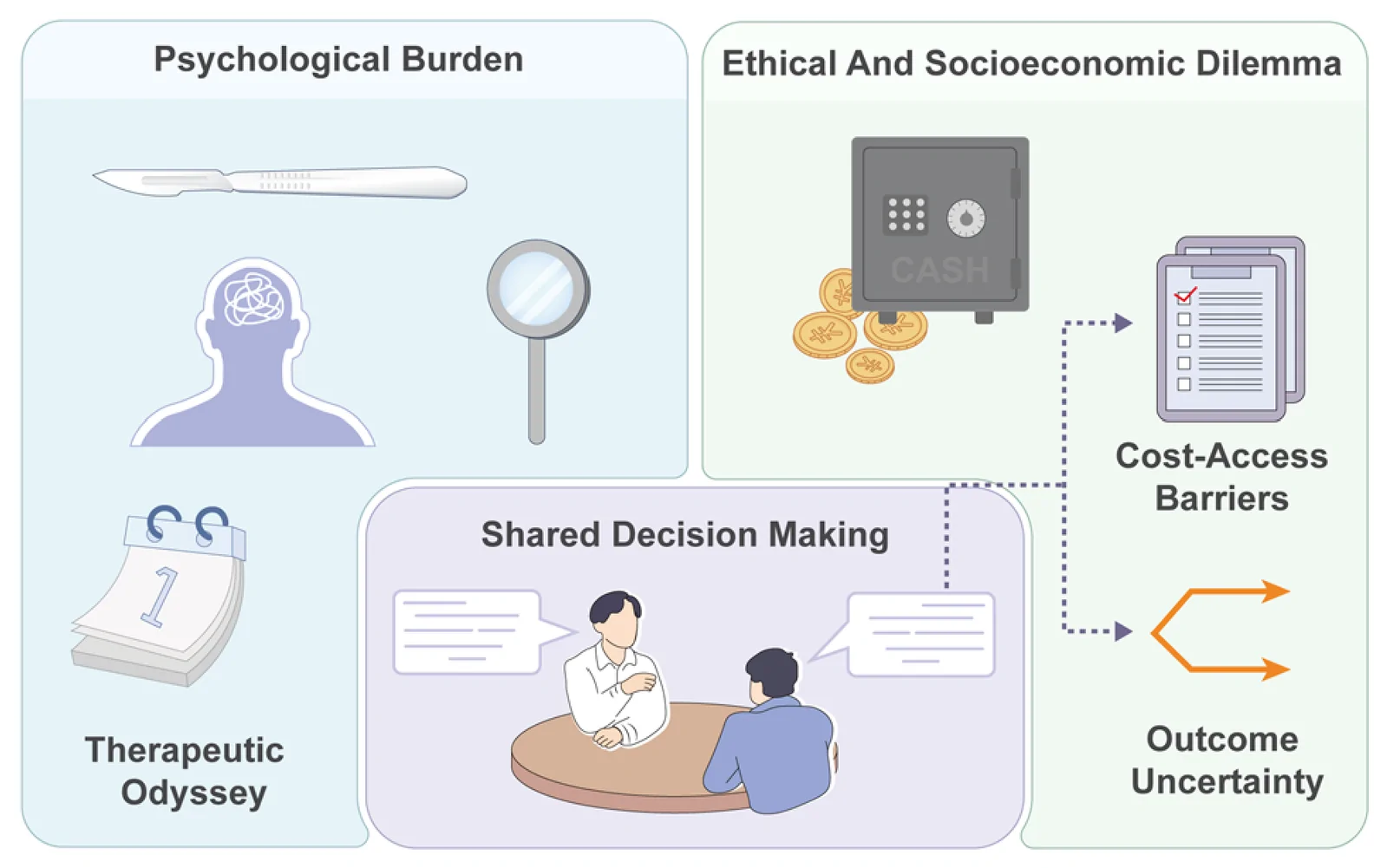
Psychological and ethical challenges in reproductive decision-making. A multidimensional array of factors can influence the decision-making processes of affected families and clinicians regarding reproductive choices and treatment pathways. Centered on the core concept of shared decision-making (SDM), the framework is primarily shaped by two interrelated domains. First, the psychological burden encompasses the initial emotional impact of diagnosis, fear of surgical interventions, psychological expectations regarding facial aesthetics following cleft repair, and the “therapeutic odyssey (the long, arduous, and often unpredictable overall therapeutic trajectory)”. Second, this psychological dimension is further compounded by ethical and socioeconomic dilemmas, which manifest as prohibitive treatment costs, cost-access barriers (limited accessibility to advanced technologies), and inherent uncertainty regarding treatment outcomes.

The acceptability of this technology varies considerably across different cultural and religious frameworks, which may significantly influence its clinical implementation and patient decision-making. In societies where the moral status of the embryo is viewed as equivalent to that of a born child, the discard of affected embryos in PGT is ethically indistinguishable from abortion. Conversely, in secular or pragmatic bioethical frameworks, the “transfer of the embryo with the best chance of a healthy life” is viewed as an act of beneficence [85]. Furthermore, in LMICs, the “double challenge” depicted in Figure 3 becomes acute: while parents may psychologically desire PGT to avoid the stigma of CLP, the prohibitive cost renders this technology a privilege of the elite. This disparity creates a psychological divide, thereby reinforcing social stratification through biological means.

### 4.2. Ethical Controversies and Shared Decision-Making Models

The advent of PGT-P has transitioned the field from the clear-cut diagnosis of lethal monogenic disorders to the murky waters of probabilistic risk reduction. This shift creates a complex ethical matrix that clinicians and patients must navigate, balancing the principles of reproductive autonomy, non-maleficence, and distributive justice [89].

The Conflict of Autonomy vs. Uncertainty: A primary ethical dilemma lies in the tension between patient autonomy and the clinical validity of the information provided. Public surveys indicate broad approval of polygenic embryo screening, but many respondents also express concern that it could foster false expectations of a healthy child [88]. However, recent qualitative studies involving healthcare professionals highlight a critical concern: PRSs are derived from population-level statistics and may not accurately predict the phenotype of an individual embryo. The “relative risk reduction” offered by PGT-P is often misinterpreted by patients as “disease elimination” [90]. Ethical conflict arises when a clinician must counsel a patient who wishes to discard a viable embryo based on a marginally elevated PRS for NSCL/P, potentially reducing the overall live birth rate of the IVF cycle for an uncertain benefit [91]. This scenario challenges the traditional boundaries of primum non nocere (first, do no harm), as the intervention (discarding embryos) may harm the patient’s chance of parenthood more than the condition it aims to prevent.

The “Slippery Slope” and Eugenics: The application of PGT-P for non-life-threatening conditions like CLP reignites debates surrounding “consumer eugenics” [92]. Critics argue that selecting against CLP contributes to the “expressivist objection” [93]. This idea is that by selecting against a disability, society expresses a negative value judgment on the lives of existing individuals with that disability. Furthermore, as PRS technology expands to include traits such as height or intelligence, the line between therapy (preventing disease) and enhancement (optimizing traits) blurs. Clinicians are increasingly finding themselves as gatekeepers [79]. If a couple requests to screen out embryos with a high risk of CLP, it could be framed as medical prevention, although its acceptability for CLP remains contested [94].

However, the underlying technology is identical to that used for non-medical trait selection. This creates a moral hazard where the medicalization of CLP risk could serve as a Trojan horse for broader, unregulated genetic selection.

To put it further, the application of PGT-P for NSCL/P raises other critical ethical concerns. We believe that an important limitation is that its clinical utility remains unvalidated and lacks robust predictive validity at the embryo stage [95]. Moreover, given effective postnatal surgical repair, we may question the ethical justification for discarding viable embryos for such limited and uncertain benefits. Third, high costs also restrict access and raise equity concerns and distributive justice debates [96]. Regulatory approaches vary widely internationally, reflecting deep ethical divisions [83]. An important point that cannot be ignored for affected families is long-term psychological risks to both offspring and parents [93,95]. Potential psychological harms have been raised because even after PRS-based selection, offspring also face the risk of illness. Parents may experience feelings of guilt and self-doubt, which can exacerbate psychological trauma and family stress. Collectively, current evidence does not support routine clinical use of PGT-P for OFC prevention [95]. Responsible future implementation demands prediction performance after repeated verification, equitable access, and comprehensive genetic counseling.

A Framework for Shared Decision-Making (SDM): Given these complexities, the traditional “non-directive” counseling model is insufficient. A robust SDM framework is essential [97]. This framework must prioritize Genetic Literacy: ensuring patients understand the difference between diagnostic testing and probability screening. Counselors must explicitly communicate that a “low-risk” score does not guarantee health, nor does a “high-risk” score guarantee disease due to environmental modifiers. Secondly, the framework must address Value Clarification: helping patients articulate why they wish to screen for CLP [97]. Is it fear of the surgery or fear of social stigma? Understanding these motivations allows for better support. Finally, informed consent in this era requires transparency about the limitations of the data, particularly for non-European populations where PRS accuracy is significantly lower. By fostering a dialogue that acknowledges both the technical limitations and the emotional weight of the decision, clinicians can help patients navigate the “double challenge” of psychological burden and ethical uncertainty.

## 5. Future Perspectives and Conclusions

The trajectory of CLP research and management is undergoing a shift—from reactive surgical reconstruction to proactive genomic prediction. There are three emerging critical frontiers: the integration of multi-omics with artificial intelligence, the refinement of clinical decision support, and the imperative for global genomic equity.

While GWASs have successfully cataloged risk loci, the “missing heritability” of NSOFC remains a significant hurdle. The future of risk prediction lies not in static DNA sequencing alone, but in the dynamic decoding of the “epigenetic–environment” interface [98]. Future research must prioritize multi-omics integration, combining genomics with transcriptomics, epigenomics, and metabolomics, to elucidate how environmental triggers (e.g., maternal nutrition and hypoxia) interact with chromatin accessibility to disrupt facial morphogenesis. Given the complexity of these high-dimensional datasets, artificial intelligence and machine learning may play a supporting role. Artificial intelligence algorithms may move beyond simple PRSs to model non-linear gene–gene interactions and recognize complex genotype–phenotype patterns that elude traditional statistical methods [99]. These tools may support future model development, but clinical utility still requires prospective validation.

It is worth noting that several critical limitations must be considered when evaluating the potential application of PGT-P for CLP. First, PRS-based embryo scores provide only a relative ranking within the limited cohort of available sibling embryos, rather than an absolute diagnostic prediction [100,101]. Second, the achievable risk reduction is highly dependent on the number of available embryos; with fewer embryos, the selection advantage diminishes substantially [79,102]. Third, PRS calibration and transferability vary considerably across ancestry groups, as current models are predominantly derived from European populations [67,103]. Fourth, pleiotropy and correlated traits may complicate selection [104], as reducing risk for one condition may inadvertently increase risk for another. Fifth, technical uncertainties (including embryo biopsy, whole-genome amplification, allelic dropout, and mosaicism) may introduce uncertainty into PGT-P results [105]. Finally, a low PRS does not eliminate the risk of CLP, given the significant environmental and epigenetic contributions to its etiology. Thus, prenatal testing and routine fetal assessment remain essential after PGT. We unanimously agree that PGT-P should remain in the research domain until rigorous prospective validation and clinical utility assessment are completed.

In clinical settings, the integration of advanced imaging and genetic profiling is a promising research direction that may enhance prenatal diagnosis, though its clinical utility remains to be established. Currently, prenatal diagnosis relies heavily on ultrasound; however, this modality has inherent limitations. For instance, evidence highlights that prenatal ultrasound cannot clearly identify subtle phenotypic variations, such as isolated palate involvement, leading to a risk of missed diagnosis [106,107]. This diagnostic blind spot underscores the necessity of complementary genetic screening. Future clinical workflows may benefit from incorporating integrated decision-support tools [99]. These digital platforms might synthesize data from high-resolution 3D ultrasound, parental genetic background, and environmental exposure history to generate a personalized “Risk Probability Profile.” Such tools might empower clinicians to move from vague empirical counseling to precise, data-driven prognostication, helping families prepare for the specific surgical and functional needs of their child.

Perhaps the most pressing challenge is the lack of diversity in genomic data. Current PRS models are predominantly trained on European cohorts, rendering them less accurate for other populations. This is a critical flaw, given that epidemiological data confirm that the prevalence of CLP varies significantly depending on race, geography, and gender [108]. Furthermore, specific phenotypic expressions, such as the laterality and location of the cleft, also demonstrate distinct prevalence rates across different genders [108]. If these population-specific nuances are ignored, the global application of PGT-P risks exacerbating health disparities—creating a system where genetic prediction works well for some ethnic groups but fails for others. Therefore, building diverse, multi-ancestry genomic biobanks represents an important research and equity priority. International data sharing could help construct reference databases that reflect the global burden of CLP, although the governance mechanisms enabling such sharing remain to be developed. In many nations, high out-of-pocket costs can substantially restrict access [109], raising the risk of a “genetic divide.” Whether public financing in high-prevalence regions (such as parts of Asia and Latin America) could improve equitable access to genetic screening is an open question for future health-services research.

While PGT-M is clinically established for families carrying a clearly actionable pathogenic variant associated with syndromic or Mendelian forms of CLP, the majority of non-syndromic CLP cases are polygenic and multifactorial, and current CLP-specific PGT-P lacks sufficient evidence of discrimination, calibration, and clinical utility. These uncertainties underscore the urgent need for future research that integrates standardized phenotyping, multi-ancestry genomic databases, functional validation of candidate loci, prospective clinical validation, and robust frameworks for risk communication and shared decision-making, thereby bridging the gap between technological promise and responsible clinical implementation. At the same time, it demands a cautious and ethical approach. Finally, the ethical issues involved also deserve particular emphasis. Unlike monogenic disorders associated with early lethality or severe disability, CLP is, in most cases, a non-lethal and surgically correctable condition, and some affected individuals generally lead normal lives after the surgery. This distinction raises a central normative question: whether, and under what circumstances, selection against embryos at risk of a treatable structural anomaly is ethically justified. At the same time, families facing the substantial surgical, speech, and psychosocial burden of CLP may reasonably seek to avoid transmitting the condition. We think navigating this tension requires shared decision-making process that weighs reproductive autonomy, the anticipated severity of the phenotype, and respect for the equal moral worth of persons with CLP.

The future of reproductive selection in CLP does not lie in the unbridled pursuit of “genetic perfection,” but in the integration of accurate molecular diagnosis, transparent risk communication, and ethical shared decision-making. By balancing technological prowess with compassionate care, we can transition from merely repairing the cleft to supporting the whole family—ensuring that every child, regardless of their genetic profile, is born into a world prepared to nurture their potential.

## Data Availability

No new data were created or analyzed in this study. Data sharing is not applicable to this article.
